# Tyrosine kinase inhibitor–associated cerebral vasculopathy with a distinct non-enhancing vessel wall phenotype: a case report

**DOI:** 10.1186/s12883-026-05192-x

**Published:** 2026-07-20

**Authors:** Jeong Eun Yang, Jae Ho Jeon, Young Rok Do, Jaeseob Yun

**Affiliations:** 1 Department of Neurology, Dong-san Hospital, Keimyung University School of Medicine, Daegu, Korea; 2 Department of Hematology and Oncology, Dong-san Hospital, Keimyung University School of Medicine, Daegu, Korea; 3 Department of Neurology, Pusan National University Hospital, Pusan National University School of Medicine and Medical Research Institute, Busan, Republic of Korea

**Keywords:** Intracranial stenosis, Vessel wall MRI, Tyrosine kinase inhibitor, Cerebrovascular disease, Case report

## Abstract

**Background:**

Tyrosine kinase inhibitors (TKIs) are associated with adverse vascular events, including cerebrovascular stenosis. However, most reported cases rely on luminal imaging, and the arterial wall characteristics of TKI-associated vasculopathy remain insufficiently characterized.

**Case presentation:**

A 47-year-old man with chronic myeloid leukemia developed recurrent transient ischemic attacks during long-term exposure to multiple tyrosine kinase inhibitors, including sequential treatment with nilotinib and ponatinib. Neurovascular imaging revealed extensive multifocal steno-occlusive lesions involving both intracranial and extracranial arteries, accompanied by severe hemodynamic compromise. High-resolution vessel wall MRI demonstrated diffuse circumferential wall thickening with negative remodeling in both distal internal carotid arteries and eccentric thickening along the anterior walls of the bilateral M1 segments of the middle cerebral artery. Notably, no definite mural enhancement was observed. Following discontinuation of ponatinib and transition to alternative therapy, cerebral perfusion improved at two months, and the patient remained clinically stable without recurrent ischemic events.

**Conclusions:**

This case illustrates a potentially distinct vessel wall thickening pattern in TKI-associated vasculopathy, providing information beyond luminal stenosis. The absence of definite mural enhancement and clinical and hemodynamic improvement after treatment modification suggest a drug-related vascular component. Vessel wall imaging may aid in the evaluation of this condition and inform management strategies.

## Background

Tyrosine kinase inhibitors (TKIs) are the standard-of-care treatment for chronic myeloid leukemia (CML) and have been shown to markedly improve long-term survival outcomes [1]. However, accumulating evidence indicates that certain TKIs, particularly second- and third-generation agents such as nilotinib and ponatinib, are associated with an increased risk of vascular adverse events, including coronary artery disease and ischemic stroke [1, 2]. Recently, cerebrovascular involvement associated with TKIs has been increasingly recognized, with several reports describing multifocal arterial stenosis and distinctive clinical features [3–5]. Nevertheless, these reports have been largely descriptive and have primarily relied on luminal imaging modalities, with limited characterization of arterial wall pathology. Consequently, management has largely followed conventional atherosclerotic paradigms, typically involving antiplatelet therapy or revascularization [5].

High-resolution vessel wall imaging (VWI) MRI provides an opportunity to characterize arterial wall pathology beyond luminal stenosis. Although a limited number of studies have explored VWI findings in TKI-associated vasculopathy [6–8], the spectrum of vascular changes remains incompletely defined. Given the diverse off-target effects of BCR-ABL inhibitors, particularly with more potent agents such as ponatinib, TKI-associated vasculopathy may involve heterogeneous mechanisms that differ from conventional atherosclerosis. In this context, VWI MRI may provide insight into underlying vascular remodeling patterns and help delineate potential differences in pathophysiology.

Here, we report a case of TKI-associated cerebrovascular vasculopathy with heterogeneous concentric and eccentric vessel wall thickening without definite mural enhancement on VWI MRI, suggesting a potentially distinct arterial wall phenotype.

## Case presentation

A 47-year-old man with a 20-year history of chronic myeloid leukemia (CML) presented with a three-month history of recurrent transient neurological symptoms. He initially developed transient weakness of the left leg in May 2025. Over time, his symptoms progressively evolved to include orthostatic dizziness and transient weakness involving both the left upper and lower extremities. The frequency of these episodes gradually increased, eventually occurring multiple times per day. His prior treatment included sequential tyrosine kinase inhibitors due to inadequate molecular response, consisting of imatinib (2005–2014), followed by nilotinib until 2018, and ponatinib initiated in September 2018, with the dose maintained at 30 mg daily since June 2025. His vascular risk factors included hypertension, which was well controlled with amlodipine 5 mg daily, and a 15-pack-year smoking history. He had no diabetes mellitus or dyslipidemia and no clinical history of coronary or peripheral arterial disease.

**Fig. 1:**
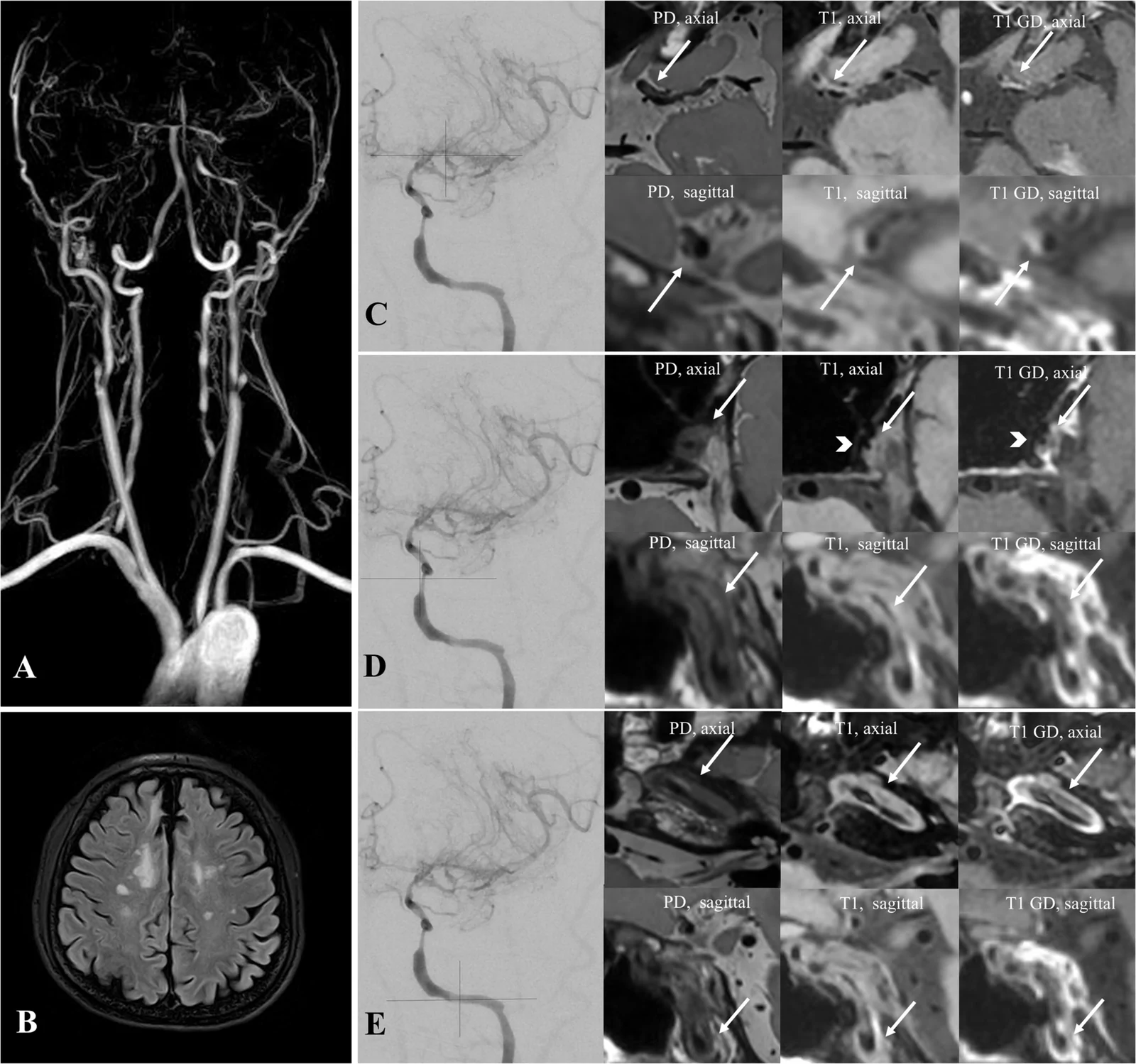
Representative angiographic and high-resolution vessel wall MRI (VWI) findings showing heterogeneous vessel wall patterns. **A** Contrast-enhanced MR angiography shows multifocal arterial stenoses, including occlusion of the right proximal internal carotid artery (ICA), severe stenosis of the left proximal ICA, and severe stenosis at the origins of both vertebral arteries. **B** Fluid-attenuated inversion recovery (FLAIR) imaging demonstrates chronic infarctions involving both frontal lobes. **C**–**E** Digital subtraction angiography (DSA) of the left ICA confirms multifocal stenoses. Corresponding VWI was obtained at the indicated levels (dotted lines). Axial images (from left to right: proton density [PD]-weighted, pre-contrast T1-weighted, and post-contrast T1-weighted sequences) with corresponding sagittal views are shown. **C** VWI at the level of the middle cerebral artery (MCA) demonstrates eccentric wall thickening along the anterior wall without definite mural enhancement (arrow). **D** VWI at the cavernous ICA level demonstrates diffuse circumferential wall thickening (arrow), most prominently on PD-weighted imaging. Severe luminal narrowing is best appreciated on T1-weighted imaging (arrowhead), without definite mural enhancement. **E** VWI at the petrous segment of the left ICA demonstrates diffuse circumferential wall thickening (arrows) without definite mural enhancement

Magnetic resonance angiography (MRA) performed in August 2025 revealed extensive multifocal steno-occlusive disease, including total occlusion of the right proximal internal carotid artery (ICA), severe stenosis of the left proximal and distal ICA, and bilateral vertebral artery (VA) orifice stenosis (Fig. 1A). Fluid-attenuated inversion recovery (FLAIR) imaging demonstrated chronic bilateral frontal lobe infarctions (Fig. 1B). Although the bilateral ICA steno-occlusive pattern initially raised suspicion for moyamoya disease, the prominent involvement of extracranial arterial segments was atypical. Genomic DNA extracted from peripheral blood was analyzed by polymerase chain reaction amplification and Sanger sequencing for the RNF213 c.14429G > A (p.R4810K) variant in exon 60; the variant was not detected. Laboratory evaluation showed a mildly elevated erythrocyte sedimentation rate (25 mm/h), while C-reactive protein, glycosylated hemoglobin, and lipid profiles were within normal limits. Screening for systemic vasculitis and autoimmune antibodies was negative. The coagulation profile was unremarkable, with a platelet count of 277,000/μl, PT-INR of 0.99, an aPTT of 28.0 s, and a fibrinogen level of 388.9 mg/dL.

**Fig. 2:**
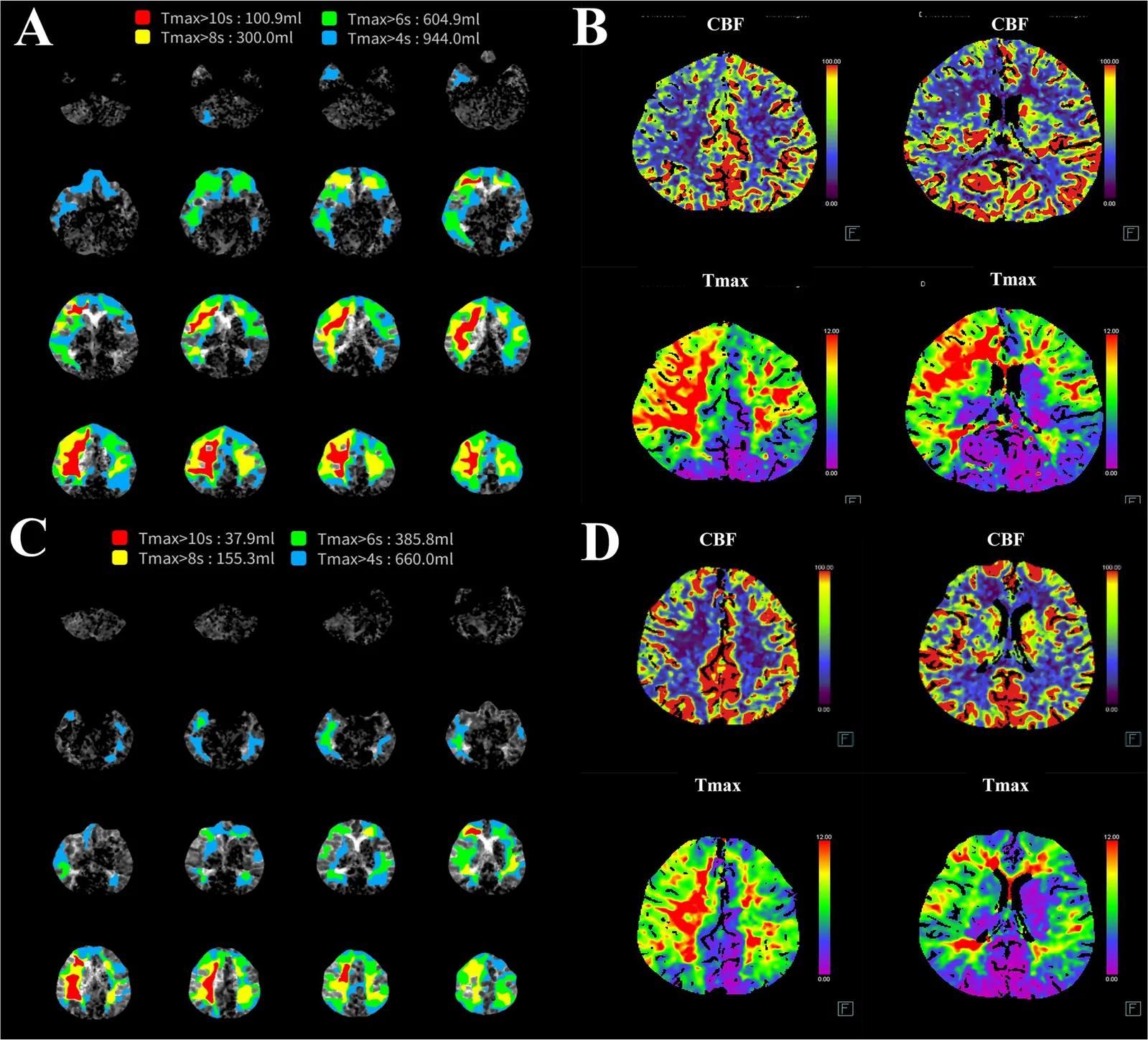
Serial perfusion CT demonstrates improvement in cerebral hemodynamics following treatment modification. **A**–**B** Baseline perfusion CT. **A** Cerebral blood flow (CBF) and Tmax maps demonstrate diffusely reduced perfusion with extensive Tmax delay, predominantly involving the bilateral anterior circulation. **B** Quantitative RAPID analysis demonstrates a markedly increased volume of hypoperfused tissue, defined by Tmax > 6 s (604.9 mL), indicating severe hemodynamic compromise. **C**–**D** Follow-up perfusion CT obtained approximately 2 months after treatment modification. **C** CBF and Tmax maps demonstrate partial restoration of cortical perfusion with reduced perfusion delay compared with baseline. **D** Quantitative RAPID analysis demonstrates a substantial reduction in hypoperfused volume, with Tmax > 6 s measuring 385.8 mL, consistent with interval improvement in cerebral perfusion

High-resolution vessel wall imaging (VWI) MRI was performed to further characterize the arterial wall pathology. The imaging demonstrated diffuse circumferential wall thickening with negative remodeling involving both distal ICAs, together with eccentric thickening confined to the anterior walls of the bilateral M1 segments of the middle cerebral arteries (MCAs). Mural enhancement was assessed qualitatively by a board-certified neuroradiologist through side-by-side visual comparison of pre- and post-contrast T1-weighted images at the same arterial segments, with reference to proton-density and pre-contrast T1-weighted images to localize the thickened arterial wall. No definite mural enhancement of the thickened arterial wall was identified, a pattern less typical of active inflammatory vasculitis (Fig. 1C–E). Baseline perfusion computed tomography (CT) demonstrated marked hemodynamic compromise, with a Tmax > 6 s volume of 604.9 mL (Fig. 2A, B).

TKI-associated vasculopathy was suspected in the context of long-term tyrosine kinase inhibitor exposure, extensive multifocal intracranial and extracranial steno-occlusive disease and lack of evidence for systemic inflammatory vasculitis. Given the temporal association, ponatinib was considered a likely contributing agent, although the contributions of prior nilotinib exposure and conventional vascular risk factors could not be excluded. Ponatinib was subsequently replaced with asciminib on August 11, 2025, and dual antiplatelet therapy (aspirin 100 mg and clopidogrel 75 mg) was initiated on August 13, 2025. At outpatient follow-up on September 10, 2025, the patient reported no recurrent transient neurological symptoms. Follow-up perfusion CT performed on October 28, 2025, showed partial improvement, with the Tmax > 6 s volume reduced to 385.8 mL (Fig. 2C, D). Brain MRI performed on October 29, 2025, showed no new infarction. During the six-month follow-up, the patient remained clinically stable without recurrence of transient ischemic attacks (TIAs) or positional symptoms.

## Discussion

This case describes multifocal intracranial and extracranial steno-occlusive vasculopathy in the setting of long-term sequential tyrosine kinase inhibitor (TKI) exposure, including nilotinib and ponatinib. High-resolution VWI MRI showed diffuse concentric ICA wall thickening and eccentric MCA wall thickening without definite mural enhancement. These findings may suggest a non-inflammatory arterial wall process that differs from typical inflammatory vasculitis and conventional intracranial atherosclerosis, although the pattern is not pathognomonic. Notably, cerebral perfusion improved following treatment modification, including discontinuation of ponatinib, transition to asciminib, and initiation of dual antiplatelet therapy.

In the present case, multifocal steno-occlusive lesions were accompanied by diffuse arterial wall thickening on VWI MRI. This pattern differs from conventional intracranial atherosclerosis, which typically manifests as focal, eccentric plaque formation rather than diffuse wall involvement [9, 10]. Although the MCA lesions were eccentric, their bilateral anterior-wall distribution differed from an isolated focal plaque pattern. No definite mural enhancement was identified on qualitative comparison of pre- and post-contrast images. Although this pattern is less typical of active inflammatory vasculitis, VWI findings alone cannot establish the underlying pathology [9, 10]. This observation is consistent with a prior report in which histopathological analysis of superficial temporal artery (STA) specimens obtained during STA–MCA bypass revealed no evidence of inflammatory cell infiltration in the affected vessels [5]. Taking these findings together, TKI-associated vasculopathy in this case may involve a non-inflammatory vascular process. Given the broad off-target kinase inhibition profile of TKIs, particularly ponatinib, such vascular changes may be mediated through endothelial dysfunction rather than conventional atherosclerotic or inflammatory mechanisms.

Vascular adverse effects differ across TKI classes. Whereas VEGF/VEGFR inhibitors are more commonly associated with hypertension, hemorrhage, aneurysm formation, and arterial dissection, BCR-ABL inhibitors such as nilotinib and ponatinib have been particularly associated with arterial occlusive events, including reported cases of progressive stenotic vasculopathy [2, 5, 11]. The heterogeneous pattern of concentric and eccentric arterial wall thickening observed in this case may reflect the cumulative vascular effects of sequential TKI exposure, including ponatinib. To date, reports incorporating vessel wall imaging have primarily involved patients treated with nilotinib (Table 1). Nilotinib has been associated with endothelial toxicity and metabolic abnormalities that may contribute to accelerated atherosclerosis [12, 13], as reflected in prior VWI studies demonstrating eccentric or mixed wall thickening patterns, often resembling plaque-like lesions [7, 8]. Notably, intraoperative findings in prior reports have shown focal, plaque-like wall thickening consistent with atherosclerosis, supporting an atherogenic process [8]. In contrast, ponatinib has a broader multi-kinase inhibition profile and may promote vascular injury through endothelial dysfunction, platelet and leukocyte activation, TNF-related endothelial signaling, oxidative stress, and plaque inflammation [12, 14, 15]. In this context, the diffuse ICA wall thickening observed on VWI MRI in our case may reflect vascular remodeling related to ponatinib-associated off-target effects rather than isolated focal plaque formation or thrombotic occlusion [12, 14]. TKI-associated arterial toxicity is not confined to intracranial vessels and has also been reported in coronary, peripheral, and extracranial cerebrovascular beds [2, 15]. In the present patient, proximal ICA and vertebral artery origin involvement indicated extracranial disease; however, more widespread systemic vasculopathy could not be assessed because dedicated systemic arterial imaging was not performed.

**Table 1:** Reported vessel wall MRI findings in TKI-associated cerebrovascular disease

Author (year)	Age/Sex	TKI (duration)	Vessel involved	Vessel wall MRI phenotype	Treatment	Outcome
Suzuki et al. [6] (2019)	55/M	Nilotinib (3 years)	Right ICA, bilateral MCA	Diffuse concentric MCA wall thickening	Dual antiplatelet therapy followed by STA-MCA bypass; nilotinib discontinuation	Symptomatic improvement after bypass surgery
Corrêa et al. [7] (2023)	67/M	Nilotinib (3 years)	Right MCA	Eccentric MCA thickening with enhancement	Nilotinib discontinuation, anticoagulant therapy	Not reported
Rai and Hara [8] (2023)	39/F	Nilotinib (3.5 years)	Right MCA, ACA, PCA, VA, left CCA	Asymmetric right MCA wall thickening	High-flow ECA-RA-M2 bypass; nilotinib discontinuation	Symptomatic improvement after bypass surgery
Present case	47/M	Ponatinib (7 years)	Both ICA, ACA, MCA, VA	diffuse concentric ICA wall thickening with eccentric MCA involvement	Ponatinib discontinuation with antiplatelet therapy	Clinical and perfusion improvement

*Abbreviations*: *ACA* anterior cerebral artery, *CCA* common carotid artery, *ECA* external carotid artery, *ICA* internal carotid artery, *MCA* middle cerebral artery, *PCA* posterior cerebral artery, *VA* vertebral artery, *RA* radial artery, *STA* superficial temporal artery, *TKI* tyrosine kinase inhibitor

An important clinical observation in this case was the improvement in neurological symptoms and cerebral perfusion following treatment modification. This finding is consistent with prior reports suggesting that TKI-associated cerebrovascular disease may exhibit a degree of clinical and hemodynamic recoverability after withdrawal of the offending agent [3]. The chronic bilateral frontal ischemic lesions involved more than one vascular territory, an atypical distribution also described in a previous report [3]. In prior reports, several patients required revascularization strategies because ischemic symptoms or hemodynamic compromise persisted despite medical management [5, 6, 8]. The present course therefore supports considering TKI withdrawal and medical therapy as an initial strategy in selected clinically stable patients. However, treatment should be individualized according to recurrent ischemic symptoms, development of new infarction, collateral status, and the severity and persistence of hemodynamic compromise. Surgical or endovascular revascularization should not be delayed when recurrent TIAs continue or progressive ischemic injury persists despite appropriate medical and oncologic treatment.

## Limitations

Several limitations should be acknowledged. This is a single case with sequential exposure to nilotinib and ponatinib, and conventional vascular risk factors may also have contributed. Pretreatment angiography and VWI were unavailable, and follow-up VWI was not performed because the patient declined due to financial concerns; no histopathological confirmation was obtained, limiting assessment of the temporal evolution and reversibility of the arterial wall changes. Because the clinical and perfusion improvement followed simultaneous ponatinib withdrawal, asciminib substitution, and dual antiplatelet therapy, and because natural recovery or hemodynamic adaptation may also have contributed.

## Conclusion

This case illustrates a potentially distinct arterial wall phenotype on VWI MRI, characterized by diffuse concentric ICA thickening and eccentric MCA wall thickening without definite mural enhancement in the setting of sequential TKI exposure, including ponatinib. VWI may complement luminal and perfusion imaging when evaluating suspected TKI-associated vasculopathy, while management should be individualized according to clinical and hemodynamic progression.

## Data Availability

The data supporting the findings of this study are available from the corresponding author upon reasonable request.

## Abbreviations

CBF: Cerebral blood flow
CML: Chronic myeloid leukemia
CT: Computed tomography
FLAIR: Fluid-attenuated inversion recovery
ICA: Internal carotid artery
MCA: Middle cerebral artery
MRA: Magnetic resonance angiography
RNF213: Ring finger protein 213
STA: Superficial temporal artery
TIA: Transient ischemic attack
TKI: Tyrosine kinase inhibitor
VA: Vertebral artery
VWI: Vessel wall imaging
